# Factors influencing caries status and treatment needs among pregnant women attending a maternity hospital in Udaipur city, India

**DOI:** 10.4317/jced.50982

**Published:** 2013-04-01

**Authors:** Santhosh Kumar, Jyothi Tadakamadla, Harish Tibdewal, Prabu Duraiswamy, Suhas Kulkarni

**Affiliations:** 1 Assistant professor. Department of Preventive Dental Sciences, College of Dentistry, Jazan University. Jazan, KSA; 2 Assistant professor. Department of Maxillofacial Surgery and Diagnostic Sciences, College of Dentistry, Jazan University. Jazan, KSA; 3 Senior lecturer. Department of Public Health Dentistry, Vidarbha Youth Welfare Society’s Dental College, Amravati. Maharashtra, India; 4 Professor, Department of public health dentistry, Sri Ramachandra Dental College, Chennai, India; 5 Professor and Head, Department of public health dentistry, Panineeya Mahavidyalaya Institute of Dental Sciences, Hyderabad, India

## Abstract

Objectives: To estimate the prevalence and severity of dental caries along with the treatment needs; to determine the factors that influence dental caries status among pregnant women attending a district maternity hospital in Udaipur, India. Study design: Study sample comprised of 206 pregnant women attending a district maternity hospital in Udaipur, India. Clinical data were collected on dental caries by DMFT and treatment needs as described in World Health Organization Dentition status and Treatment needs. Results: The overall caries prevalence was 87%. Mean caries experience differed significantly among women in various trimesters, it was found to be 3.59 and 3.00 in 1st and 2nd trimester subjects respectively while it was greatest (4.13) among those in 3rd trimester. One surface filling was the most predominant treatment need. Age and occupation of husband explained a variance of 6.8% and 4.2% for decayed and filled components respectively while the only predictor for missing teeth and DMFT that explained a variance of 9.6% and 5.7% respectively was trimester of pregnancy. Conclusions: Dental caries experience and the need for one surface restoration increased with age. Trimester of pregnancy was a significant predictor for missing teeth and DMFT, while decayed teeth and filled teeth were influenced by age and socio-economic level respectively.

** Key words:**Dental caries, treatment needs, pregnant, age, trimester.

## Introduction 

A recent review by Suresh and Radfar (1) stated that complex hormonal interactions that cause a wide range of physiologic changes are encountered during pregnancy. The levels of sex steroid hormones in saliva increase during pregnancy. In addition, pregnant women present more inflammation and gingival bleeding than the general population and this effect is related to the dental biofilm, the microbial flora and the hormonal levels (2).

In addition to gingival changes, the other manifestations associated with pregnancy include chloasma (bilateral brown patches in the midface), facial telangiectasia, sialorrhea, tooth surface loss usually related to vomiting when severe (hyperemesis gravidarum), increased mobility of teeth, changes in the severity of oral aphthae (3). 

Salvolini et al., (4) observed that several salivary changes occur during pregnancy like flow, composition, pH and hormone levels. The change in composition of the saliva includes a decrease in pH and sodium and an increase in potassium, protein and oestrogen levels. 

It has been observed that pregnant women are more susceptible for dental caries than non-pregnant women. The reasons for more dental caries in pregnant women have been detailed as increased acidity in the oral cavity, sugary dietary cravings, and inadequate attention to oral health (5). 

There is scarcity of literature from Indian subcontinent and the study region regarding the caries status and the influencing factors among pregnant women. Hence, the present study intended to estimate the prevalence and severity of dental caries and treatment needs, and to determine the factors that influence dental caries status among pregnant women attending a district maternity hospital in Udaipur, India. The data thus obtained would be helpful in planning oral health prevention and intervention programs for the study population.

## Material and Methods

The target population for this study was pregnant women attending a district maternity hospital in Udaipur district, Rajasthan, India. The study design was cross sectional and the final sample consisted of 206 pregnant women, 38 belonging to 1st trimester and 84 in 2nd and rest in their 3rd trimester. The age of women ranged from 18 to 35 years with mean age being 23.43 (SD-3.79). All the women present on the days of the survey were included in the study and those who were experiencing labour pain, along with those who were uncooperative or unwilling to give consent, comprised the exclusion criterion. The current study design is similar to our previous publication (6) on the periodontal status of the same study population. Informed written consent was obtained from all participants and ethical clearance was availed from ethical committee of Darshan Dental College and Hospital, Udaipur, India. Information on socio-demographic characteristics of the participants like age, trimester, family income, occupation and education of the subject and their spouses was procured by means of personal interviews administered by the examiner, immediately after the clinical dental inspection with each of the subjects. All the subjects were examined in a mobile dental unit, caries status was recorded using the DMFT index and need for dental treatment was assessed according to WHO Dentition status and Treatment Needs as was done in previous studies (7,8).

Mouth mirrors and Community Periodontal Index probes were used for clinical examination by a single examiner assisted by a recording clerk. The intra examiner consistency for caries assessment was 93.2%. Data was entered into spreadsheets and was subjected to statistical analysis by SPSS (statistical package social sciences), version 15.0. Means and standard deviations were assessed and one way ANOVA was executed for comparing various components of DMFT index in relation to age groups and trimester of pregnancy. Step wise multiple linear regression analysis was executed to estimate the linear relationship between dependent variables (Decayed teeth, Missing teeth, Filled teeth and DMFT) and various independent variables (age, trimester, family income, occupation and education of the subject and their spouses). The stepwise multiple linear regression analysis examines the variables in the block at each step for entry or removal. The variables were entered or removed from the model depending on the significance (probability) of the F value.

## Results

 Table 1 depicts that overall caries prevalence was 87%. Caries prevalence different significantly between the various age groups, all the subjects belonging to 18-22 and 32-36 age groups had at least one carious tooth. Statistical analysis revealed significant differences between the trimesters of pregnancy with the prevalence gradually increasing with the trimester.

**Table 1:**
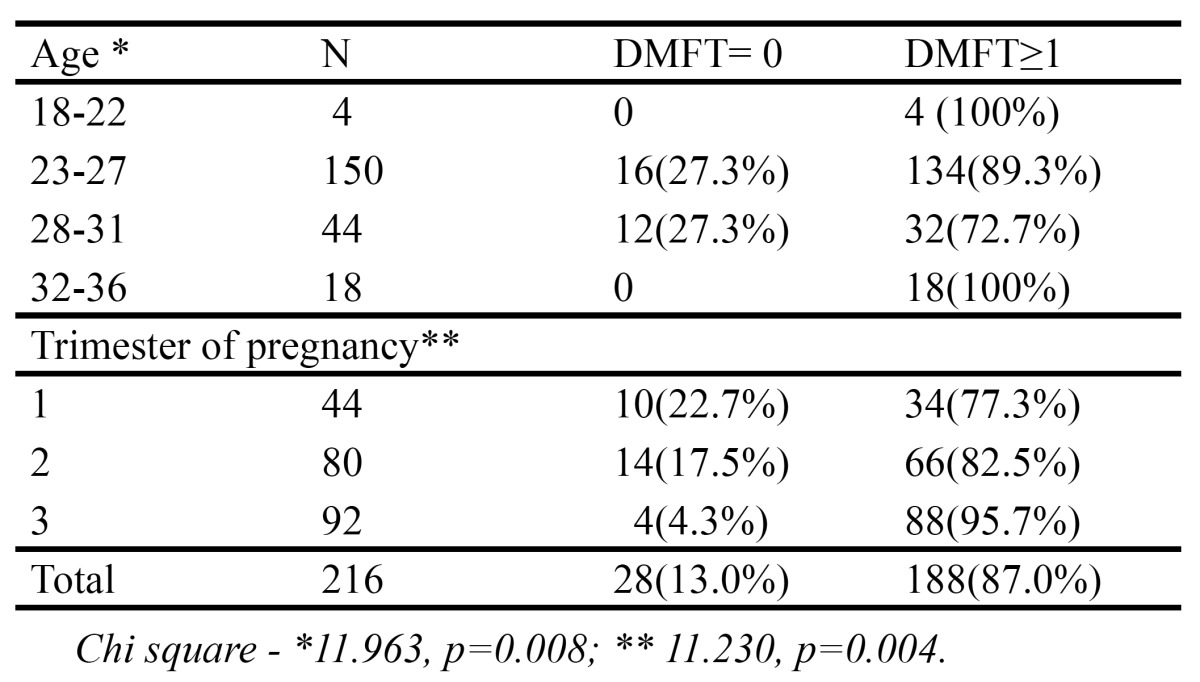
Percentage distribution of dental caries in pregnant according to age and trimester.

The mean caries experience by age group and trimester of pregnancy is presented in Table 2 and  Table 3 respectively. Caries experience of the entire study population was 3.60. Decayed component was the dominant expression of caries experience with a mean of 3.34 (92.7%). The youngest age group experienced lowest caries (3.00) while it was highest (5.11 DMFT) in the oldest age group. Significant differences were noticed between the trimesters of pregnancy for mean decayed component and DMFT. Mean caries experience differed significantly among women in various trimesters, it was found to be 3.59 and 3.00 in 1st and 2nd trimester subjects respectively while it was greatest (4,13) among those in 3rd trimester.

**Table 2:**
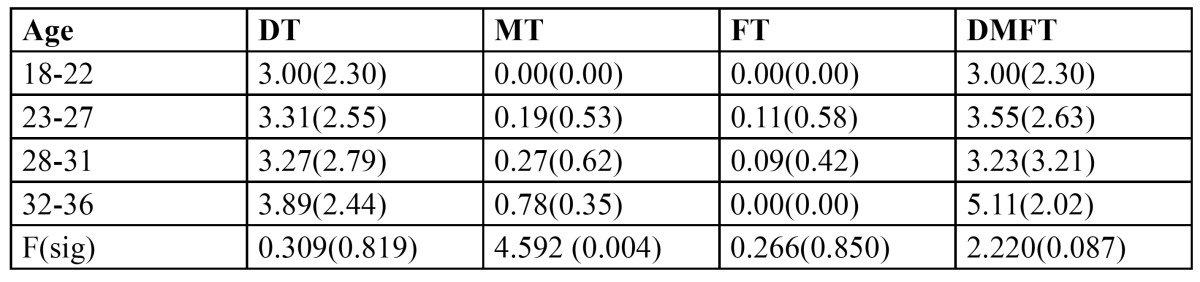
Distribution of number of decayed, missing, filled and DMFT per woman in relation to age group.

**Table 3:**
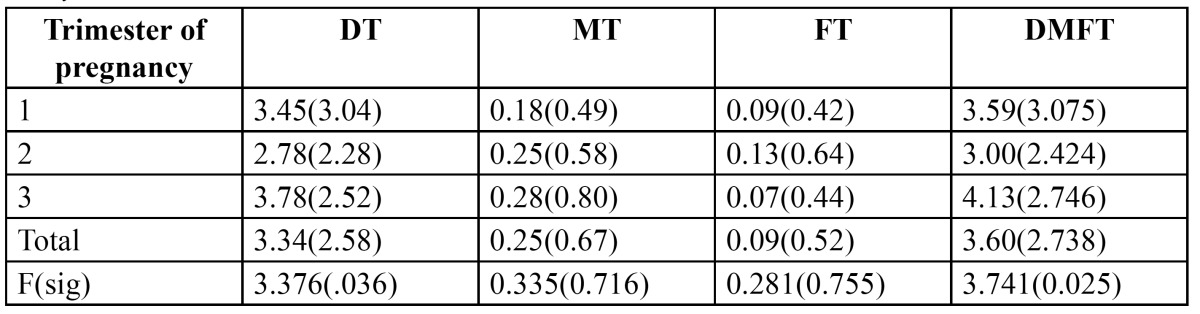
Mean decayed, missing, filled and DMFT per woman in relation to trimester of pregnancy.

 Table 4 and  Table 5 illustrate treatment needs in various age groups and trimesters respectively. One surface filling was the most predominant treatment need, the mean number of teeth that required one surface restoration per woman was 2.96. However, the need for other treatment procedures was minor with 0.16 teeth requiring fissure sealant and 0.14 requiring crown.

**Table 4:**
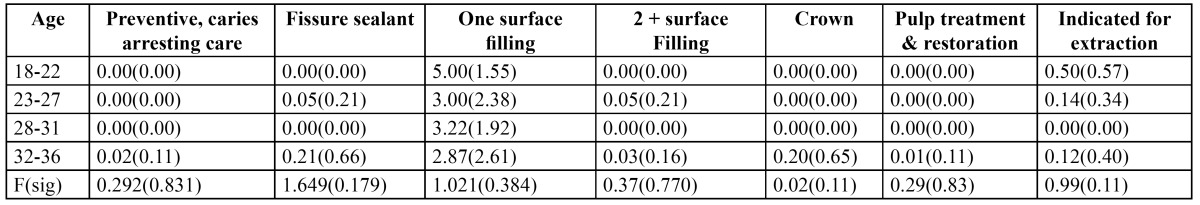
Mean number of teeth per women requiring various treatments according to age group.

**Table 5:**
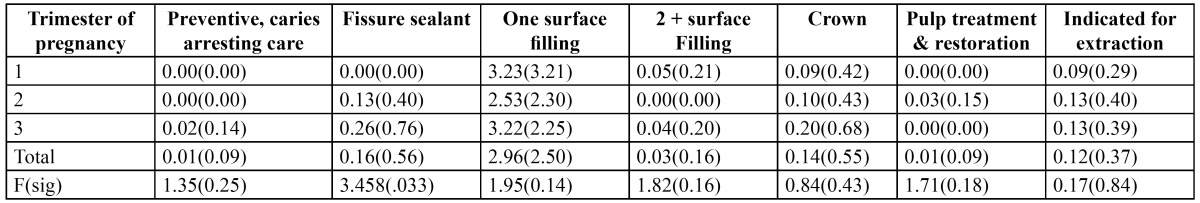
Mean number of teeth requiring various treatments in relation to the trimester of pregnancy.

In the age group 18-22 years, a mean of 5.00 was recorded for one surface filling. There were no teeth that required crown in any age group except for the oldest where a mean of 0.2 was observed. Though only 0.01 teeth required pulp care and restoration, the need was observed only among the oldest age group.

Alike the caries experience, teeth requiring single surface filling decreased among the women of second trimester followed by an increase in the subjects in their third trimester. The need for fissure sealant increased with the trimester and the teeth requiring caries arresting care was only observed in individuals belonging to third trimester of pregnancy. The need for crown and extraction was greater for women in third trimester in comparison to those in first trimester.

Step wise linear regression analysis was executed to assess the influence of various socio demographic variables on caries experience as depicted in table 6. The only predictor for decayed teeth that explained a variance of 9.6% was age whereas trimester of pregnancy and occupation of husband explained a variance of 6.8% and 4.2% for missing and filled components respectively. However the single predictor for the total caries experience (DMFT) was trimester of pregnancy which had an influence of 5.7%. The other variables which were non significant were excluded from the analysis.

## Discussion

The results of the present study could not be generalized to the pregnant women of the whole nation because of the limitations in sampling procedure. Caries experience for the entire study population was 3.60 in contrast to a whopping 12.57 among post-partum mothers of south east Hungary reported by Radnai et al., (9) On the other hand, a recent study (10) among pregnant women attending a rural teaching hospital in India reported compara-ble DMFT of 4.08. Moreover, Soderling et al., (11) reported a mean DMFT of 18 among expectant mothers in Finland and a mean DMFT of 14 was observed among pregnant women of Brazill by Zanata et al., (12). The vast difference in the caries experience between the present study population and the previous studies could be attributed to the regional and socio-cultural differences between the regions.

Decayed component contributed for 92.7% of the total caries experience and thus untreated dental caries constituted for a major proportion of the total caries experience. The youngest age group experienced lowest mean while it was highest in the oldest age group. The increase in caries experience with the increase in age can be ascribed to accumulated untreated caries in accordance to a previous study (13).

Caries experience among women in second trimester was lesser than those in their first trimester moreover it was 1.13 DMFT greater among women in their third trimester than subjects belonging to first trimester. A past study by Papp et al., (14) observed that caries experience increased with progressing pregnancy.

Similar observations were found for the need of one surface restoration. One surface restoration was the most required treatment in the study population and the need decreased as the age advanced, this is in agreement with the findings of Jago et al., (15).

The need for fissure sealant increased with the trimester and the teeth requiring caries arresting care was only observed in individuals belonging to third trimester of pregnancy which could be attributed to the rise in oestrogen level with the trimester.

Shafer and Mahler (16) demonstrated that diethylstilbestrol and estradiol are responsible for increased dental caries development on experimental rats. Their data showed that androgens are without effect on dental caries development in either male or female rats, while oestrogens increase dental caries significantly.

It is evident from the present study that the decayed component was significantly related to age in accordance to a previous study among post partum mothers in south east Hungary (9).

However another study reported that the DMFT index was not dependent on education level, profession and place of residency of pregnant women. The only predictor that influenced filled component was occupation of husband, the reasonable explanation for this observation is that the women whose husbands are in high profession would afford for dental treatment more than those in low professions. Though, occupation of the pregnant women was assessed it failed to show any influence as most of the subjects were house wives.

In conclusion, the present study revealed that dental caries experience and the need for one surface restoration increased with age. Moreover, caries experience was greater among women in third trimester than among subjects in their first trimester of pregnancy. Trimester of pregnancy was a significant predictor for filled teeth and DMFT, while decayed teeth and filled teeth were influenced by age and socio-economic level respectively. Future studies on larger samples covering a wider area are anticipated that would aid in planning oral health preventive and promotive programs for the present study population.
